# Mesh Exposure After Laparoscopic Sacrocolpopexy with Concomitant Total Hysterectomy or Uterine Preservation: A Retrospective Cohort Study

**DOI:** 10.1007/s00192-026-06554-2

**Published:** 2026-03-17

**Authors:** Rusha Yin, Yiwei Zhang, Ying Xiao, Juan Chen, Lan Zhu

**Affiliations:** 1 Department of Obstetrics and Gynecology, State Key Laboratory of Common Mechanism Research for Major Diseases, National Clinical Research Center for Obstetric & Gynecologic Diseases, Peking Union Medical College Hospital, Chinese Academy of Medical Sciences & Peking Union Medical College, Beijing, 100730 China; 2 Department of Gynecology, Beijing Shunyi Maternal and Child Health Hospital, Beijing, 101300 China

**Keywords:** Hysterectomy, Laparoscopic sacrocolpopexy, Mesh exposure, Uterine preservation

## Abstract

**Introduction and Hypothesis:**

Although the role of uterine preservation during open sacrocolpopexy was well-explored, data regarding laparoscopic sacrocolpopexy (LSC) are controversial. This study was aimed at comparing the risk of mesh exposure between LSC with total hysterectomy and the uterine-preserving approach.

**Methods:**

This retrospective cohort study included 198 patients who underwent LSC for pelvic organ prolapse. Based on surgical approach, patients were divided into two groups: those with concomitant total hysterectomy (*n* = 169) and those who underwent uterus-preserving LSC (*n* = 29). The primary outcome was mesh exposure. A logistic regression model was employed to calculate odd ratios (OR) and 95% confidence interval (CI) while adjusting potential confounding factors. Secondary outcomes included the composite success, anatomical outcomes, and self-reported symptom improvement.

**Results:**

The median follow-up time of the whole cohort was 32 months. The incidence of mesh exposure was higher in the hysterectomy group compared with the uterine preservation group (27 out of 169, 16.0% vs 1 out of 29, 3.4%, *p* = 0.13). After controlling confounding factors, concomitant hysterectomy was associated with a nearly tenfold increased risk of mesh exposure (adjusted OR, 9.9; 95% CI, 1.5 to 199.0). There was no statistically significant difference in the composite success endpoint between the two groups (107 out of 131, 81.7% vs 21 out of 25, 84.0%, *p* = 0.99).

**Conclusions:**

Concomitant hysterectomy during LSC was associated with increased risk of mesh exposure, and the composite success rates of the two groups were similar.

**Supplementary Information:**

The online version contains supplementary material available at 10.1007/s00192-026-06554-2

## Introduction

Pelvic organ prolapse (POP) is common in postmenopausal women and adversely affects their quality of life. It is expected that 12% of women will undergo surgery for POP in their lifetime [1]. In China, the prevalence of symptomatic POP was approximately 9.6% [2]. Surgery is an effective treatment. A 2023 Cochrane review compared different surgical methods and strongly supported sacrocolpopexy (SC) as the gold standard for apical prolapse based on superior rates of anatomical success, decreased patient awareness of prolapse, and less of a need for repeat surgeries [3, 4]. Nowadays, with the development of minimally invasive techniques, laparoscopic sacrocolpopexy (LSC) or robotics-assisted sacrocolpopexy has been widely used for its benefits of less blood loss and faster recovery while yielding similar anatomical and functional outcomes [5, 6]. Despite the high success rate of SC, the mesh-related complications introduced by synthetic mesh still remain a concern. The rate of mesh exposure after SC varies from 3% to 10% [7], and is influenced by the type of mesh [8], the characteristics of the population [8–10], surgical techniques used, and the duration of follow-up.

Whether concurrent hysterectomy increases the risk of mesh exposure remains a controversial issue. Meriwether et al. compared hysterectomy plus SC with sacrohysteropexy in a meta-analysis and reported reduced risk of mesh exposure and similar objective success rate in women with uterine-preserving surgery [11]. However, both the open approach and the laparoscopic approach were included in their study. A recent meta-analysis compared laparoscopic total or supracervical hysterectomy with uterine preservation during LSC, and the results showed that concurrent hysterectomy was not associated with a higher rate of mesh-related complications but was associated with higher objective and subjective success rates [12]. This meta-analysis included heterogenous studies with short follow-up duration, small sample size, and multiple cofounding factors. Whether LSC should be combined with hysterectomy still deserves further research.

The primary objective of this study was to compare the mesh exposure rate between women with total hysterectomy and those with uterine preservation during LSC. The secondary objective was to compare the success rates of the two groups.

## Materials and Methods

### Study Population

This was a retrospective cohort study, enrolling 238 women who received LSC for symptomatic POP between February 2013 and September 2024. The study has been approved by the Ethics Committee. Patients who had undergone total or subtotal hysterectomy previously, lacked key information, or had uterine or vaginal malformation were excluded. Those with no available data from postoperative follow-up assessments were also excluded.

### Surgical Procedure

All surgical procedures were performed by two experienced senior urogynecologists. The decisions to preserve or remove the uterus was made through shared decision-making with the patients, in light of the uterine condition and the patients’ preferences. For patients with concurrent hysterectomy, total hysterectomy was performed via a laparoscopy-assisted vaginal route or a laparoscopy route. Next, modified LSC was performed [13]. The operation was divided into two stages. During the vaginal surgery, hydro-dissection was performed to create two newly dissected anterior and posterior vaginal walls, each 3.5cm by 3 cm, close to the vaginal vault. Then, two precut polypropylene mesh was secured to these dissected vaginal walls with interrupted nonpenetrating sutures. Laparoscopically, the mesh was fixed to the anterior longitudinal ligament inferior to the sacral promontory with 2–0 non-absorbable sutures (Ethicon).

For patients with uterine preservation, the mesh was trimmed into a rectangular shape for the posterior compartment and a T-shape for the anterior compartment (Supplemental Fig. 1). The anterior mesh was secured to the anterior vaginal wall with interrupted non-absorbable sutures, ensuring that the sutures did not penetrate the mucosal layer. The two lateral arms were then tunneled through an avascular area of the broad ligaments. The posterior mesh was fixed to the posterior vaginal wall and the pericervical ring with similar sutures. Finally, the two arms of the anterior mesh were anchored obliquely to the pericervical ring and the posterior vaginal wall, where they were sutured to the posterior mesh.

Two types of mesh (Gynemesh, Ethicon, USA, and Tiloop, 10*15cm, pfm medical, Cologne, Germany) were used in this cohort. Vaginal estrogen was prescribed twice weekly for postmenopausal women and for those entering surgical menopause following bilateral oophorectomy.

### Data Collection and Follow-Up

The medical records of patients who underwent LSC were reviewed by two independent researchers. The demographic data, medical history, preoperative POP Quantification (POP-Q), surgery details, and perioperative and postoperative complications were collected.

Patients were advised to attend follow-up visits at 3 months postoperatively and annually thereafter. At each visit, they underwent a detailed medical interview and a pelvic examination in the clinic. Any patient-reported discomfort and identified mesh exposure would be recorded. In addition, patients were asked to complete questionnaires, including the 20-item Pelvic Floor Distress Inventory (PFDI-20) [14], the seven-item Pelvic Floor Impact Questionnaire (PFIQ-7) [15], and the 12-item Pelvic Organ Prolapse/Urinary Incontinence Sexual Function Questionnaire (PISQ-12) [16]. Patients were also asked to rank their satisfaction using the Patient Global Impression of Improvement (PGI-I) questionnaire.

### Outcome Measures

Primary outcome was mesh exposure, which was defined as the visualization of vaginal mesh through separated vaginal epithelium [17]. Secondary outcomes included the composite success, anatomical outcomes, and self-reported symptom improvement. The definition of composite success must meet the following three criteria:

1. No vaginal bulge symptoms, indicated as a negative response of “none” to question 3 of PFDI-20
2. No POP-Q point beyond the hymen
3. No retreatment for POP (including repeated surgery, pessary use, and pelvic floor physical therapy) [18]

### Statistical Analysis

All data were analyzed using R software (R Foundation for Statistical Computing, Vienna, Austria). The post hoc analysis showed that we had 81.9% power to detect the difference from 16.0% in the hysterectomy group to 3.4% in the uterine preservation group based on an alpha of 0.05. Normally distributed data were presented as mean with standard deviation (SD) and were compared using a *t* test. Non-normally distributed data were presented as medians with quantiles and were compared using a nonparametric test. Categorical data were presented as numbers with percentages and were compared using Chi-squared test or Yate’s continuity correction. All tests were two-sided, with significance set at *p* < 0.05. The logistic regression model was performed to calculate odds ratio (OR) and 95% confidence interval (CI), and potential cofounding factors were adjusted.

## Results

A total of 238 patients who underwent LSC for POP between February 2013 and September 2024 were assessed. The following patients were excluded: those with previous total or subtotal hysterectomy (*n* = 27), lack of key information (*n* = 3), lost to follow-up (*n* = 7), or with uterine/vaginal malformation (*n* = 3). Consequently, 198 patients were finally included in the analysis, and the median follow-up time was 32 (interquartile range, 11 to 60) months. Among them, 169 underwent concurrent hysterectomy, whereas 29 preserved the uterus (Supplemental Fig. 2).

The basic characteristics of the two groups are shown in Table 1. Women in the uterine preservation group were younger (40 ± 6.0 vs 53 ± 6.2, *p *< 0.01). In addition, there were fewer women with hypertension (0 vs 37 out of 169, 21.9%, *p* = 0.01) in the uterine preservation group. The length of the perineal body in the uterine preservation group was slightly longer (3.6 ± 0.7 vs 3.2 ± 0.8, *p* = 0.01). Other characteristics were similar in the two groups. As for the surgery details, the operative time of the uterine preservation group was approximately 138 min, similar to that in the hysterectomy group (138.6 ± 30.4 vs 134.5 ± 37.2, *p* = 0.58, Table 2). In addition, there were no significant differences in estimated blood loss, catheterization time, hospital stays, concurrent surgery, or postoperative complications between the two groups.

**Table 1:** Basic characteristics of the two groups

Characteristics	Uterine preservation (*n* = 29)	Hysterectomy (*n* = 169)	*p* value
Age, mean ± SD, years	40 ± 6.0	53 ± 6.2	<0.01*
BMI, mean ± SD, kg/m^2^	24.3 ± 2.8	24.8 ± 3.1	0.42
Gravity, median (IQR)	3 (2, 3)	3 (2, 4)	0.64
Vaginal parity, median (IQR)	2 (1, 2)	1 (1, 2)	0.15
Diabetes, *n* (%)	0	16 (9.5)	0.17
Hypertension, *n* (%)	0	37 (21.9)	0.01*
Urinary incontinence, *n* (%)	4 (13.8)	42 (24.9)	0.29
Allergy history, *n* (%)	1 (3.4)	33 (19.5)	0.06
Prior POP surgery, *n* (%)	1 (3.4)	9 (5.3)	0.99
POP-Q stage, *n* (%)			0.51
3	24 (82.8)	127 (75.1)	
4	5 (17.2)	42 (24.9)	
POP-Q measurements, mean ± SD, cm			
Aa	1.1 ± 1.0	1.5 ± 1.1	0.08
Ba	3.3 ± 1.7	3.9 ± 1.8	0.13
C	4.2 ± 1.5	4.2 ± 2.0	0.96
GH	5.7 ± 0.9	5.4 ± 0.9	0.15
PB	3.6 ± 0.7	3.2 ± 0.8	0.01*
TVL	8.0 ± 0.4	8.0 ± 0.5	0.85
Ap	−0.8 ± 1.3	−0.7 ± 1.5	0.62
Bp	−0.2 ± 2.4	0.2 ± 2.6	0.45
D	−1.6 ± 3.0	−1.3 ± 3.2	0.54
Self-reported symptom scores, mean ± SD			
PFDI-20	70.6 ± 50.1	69.0 ± 52.8	0.88
POPDI	33.8 ± 17.3	36.8 ± 21.0	0.49
UDI	21.8 ± 19.1	26.1 ± 22.3	0.35
CRADI	17.7 ± 22.5	11.5 ± 16.3	0.09
PFIQ-7	39.4 ± 43.4	45.2 ± 43.4	0.52
POPIQ	22.7 ± 24.8	23.8 ± 21.2	0.81
UIQ	11.2 ± 16.3	16.0 ± 20.3	0.24
CRAIQ	5.5 ± 10.0	5.4 ± 11.0	0.95
PISQ-12^a^	30.3 ± 6.9	28.9 ± 6.7	0.41
Follow-up time, mean (SD), month	27 (12, 45)	35 (11, 60)	0.91

*BMI* body mass index, *POP* pelvic organ prolapse, *POP-Q* Pelvic Organ Prolapse Quantification, *GH* genital hiatus, *PB* perineal body, *TVL* total vaginal length, *PFDI-20* 20-item Pelvic Floor Distress Inventory, *POPDI* Pelvic Organ Prolapse Distress Inventory, *UDI* Urinary Distress Inventory, *CRADI* Colorectal–Anal Distress Inventory, *PFIQ-7* seven-item Pelvic Floor Impact Questionnaire, *POPIQ* Pelvic Organ Prolapse Impact Questionnaire, *UIQ* Urinary Impact Questionnaire, *CRAIQ* Colorectal–Anal Impact Questionnaire, *PISQ* 12-item Pelvic Organ Prolapse/Urinary Incontinence Sexual Function Questionnaire, *SD* standard deviation, *IQR* interquartile range

*p < 0.05

^a^20 women in the uterine preservation group and 115 women in the hysterectomy group had an active sexual life

**Table 2:** Perioperative details and complications between the two groups

	Uterine preservation (*n* = 29)	Hysterectomy (*n* = 169)	*p* value
Operative time, mean ± SD, min	138.6 ± 30.4	134.5 ± 37.2	0.58
Estimated blood loss, mean ± SD, ml	65.2 ± 67.9	79.2 ± 49.3	0.18
Catheterization, mean ± SD, day	1.5 ± 1.1	1.3 ± 0.8	0.27
Hospital stays, mean ± SD, day	5.1 ± 3.1	5.1 ± 2.6	0.93
Concurrent surgery, *n* (%)			
Anti-incontinence	4 (13.8)	21 (12.4)	0.99
Anterior vaginal wall repair	0	11 (6.5)	0.33
Posterior vaginal wall repair	1 (3.4)	5 (3.0)	0.99
Mesh type			0.15
Gynemesh	13 (44.8)	103 (60.9)	
Tiloop	16 (55.2)	66 (39.1)	
Postoperative complications, *n* (%)			
Infection	1 (3.4)	7 (4.1)	0.99
Hemorrhage	1 (3.6)	6 (3.6)	0.99
Bladder injury	–	–	–

*SD* standard deviation

Mesh exposure occurred in only one case in the uterine preservation group. This incidence was considerably lower than that in the hysterectomy group, in which 27 out of 169 patients were affected (3.4% vs 16.0%, *p* = 0.13). The single case of mesh exposure in the uterine preservation group presented with vaginal bleeding (without pain) and the exposed mesh, located at the vaginal apex and approximately 1 cm in size, was finally trimmed in the outpatient clinic. Although in the hysterectomy group, 10 patients were asymptomatic, 12 presented with vaginal bleeding, 3 reported vaginal pain, and 2 patients reported both vaginal bleeding and pain. The exposed mesh in the hysterectomy group was entirely located at the vaginal apex. Most of exposed mesh was less than 1 cm (20 out of 27, 74.1%), and was trimmed in the outpatient clinic (25 out of 27, 92.6%). After adjusting for potential cofounding factors, concomitant hysterectomy was a risk factor of mesh exposure after LSC (adjusted OR 9.9, 95% CI 1.5 to 199.0, *p* = 0.04, Table 3).

**Table 3:** Unadjusted and adjusted logistic regression analyses between concomitant hysterectomy and mesh exposure

Models	OR (95% CI)	*p* value
Unadjusted model	5.3 (1.1 to 96.9)	0.11
Adjusted model 1^a^	9.9 (1.5 to 198.2)	0.04*
Adjusted model 2^b^	9.9 (1.5 to 199.0)	0.04*

*OR* odds ratio, *CI* confidence interval

*p < 0.05

^a^Adjusted for age and mesh type.

^b^Adjusted for age, mesh type, and variables with *p* value less than 0.05, including hypertension, and the length of PB

There were 25 patients in the uterine preservation group and 131 patients in the hysterectomy group completing postoperative questionnaires and they were included in secondary outcome analyses (Table 4). The composite success endpoint was achieved by 21 out of 25 patients (84%) in the uterine preservation group, a proportion comparable with that in the hysterectomy group (107 out of 131, 81.7%, *p* = 0.99). There was no significant difference in anatomical success. In addition, the self-reported symptom improvement after surgery did not show obvious differences between the two groups.

**Table 4:** Anatomical success and subjective improvement in the two groups

Outcomes	Uterine preservation (*n* = 25)	Hysterectomy (*n* = 131)	*p* value
Composite success, *n* (%)	21 (84.0)	107 (81.7)	0.99
Anatomical success	24 (96.0)	128 (97.7)	0.99
Subjective success	21 (84.0)	108 (82.4)	0.99
Retreatment for POP	0	0	–
POP-Q points, mean ± SD, cm			
Aa	−1.3 ± 1.0	−1.8 ± 1.0	0.03*
Ba	−1.3 ± 1.0	−1.8 ± 1.1	0.07
C	−7.6 ± 1.6	−8.3 ± 1.0	0.01*
GH	5.3 ± 1.0	5.1 ± 1.0	0.55
PB	3.6 ± 0.8	3.5 ± 0.7	0.44
TVL	8.2 ± 0.6	8.5 ± 0.7	0.09
Ap	−2.6 ± 0.4	−2.1 ± 0.9	0.01*
Bp	−2.6 ± 0.4	−2.1 ± 0.9	0.01*
D	−7.7 ± 1.3	–	–
Self-reported symptom improvement, mean ± SD^a^			
PFDI-20	−34.9 ± 44.6	−28.0 ± 64.2	0.62
POPDI	−24.5 ± 17.4	−22.6 ± 24.1	0.72
UDI	−8.5 ± 17.3	−7.5 ± 29.4	0.86
CRADI	−5.0 ± 15.6	−3.1 ± 18.8	0.64
PFIQ-7	−21.8 ± 31.9	−29.2 ± 49.4	0.49
POPIQ	−17.0 ± 21.9	−18.4 ± 23.2	0.79
UIQ	−1.5 ± 13.3	−8.9 ± 22.0	0.12
CRAIQ	−3.3 ± 8.5	−2.0 ± 13.9	0.66
PISQ-12			
Before surgery^b^	30.4 ± 6.3	28.7 ± 6.8	0.35
After surgery^c^	31.8 ± 10.0	30.4 ± 7.3	0.52
PGI-I response of much better or very much better, *n* (%)	23 (92.0)	117 (91.4)	0.99

*GH* genital hiatus, *PB* perineal body, *TVL* total vaginal length, *PFDI-20* 20-item Pelvic Floor Distress Inventory, *POPDI* Pelvic Organ Prolapse Distress Inventory, *UDI* Urinary Distress Inventory, *CRADI* Colorectal–Anal Distress Inventory, *PFIQ-7* seven-item Pelvic Floor Impact Questionnaire, *POPIQ* Pelvic Organ Prolapse Impact Questionnaire, *UIQ* Urinary Impact Questionnaire, *CRAIQ* Colorectal–Anal Impact Questionnaire, *PISQ* 12-item Pelvic Organ Prolapse/Urinary Incontinence Sexual Function Questionnaire, *PGI-I* Patient Global Impression of Improvement

*p < 0.05

^a^The symptom improvement score is the difference between the postoperative and preoperative scores

^b^17 women in the uterine preservation group and 89 women in the hysterectomy group had an active sexual life

^c^16 women in the uterine preservation group and 54 women in the hysterectomy group had an active sexual life

## Discussion

In this study, we retrospectively compared the postoperative outcomes of women who underwent LSC with concurrent hysterectomy and those with uterine preservation, and the results showed that concomitant hysterectomy was a risk factor for mesh exposure after LSC.

In our cohort, women with concomitant hysterectomy were older, which aligns with the clinical reality. After adjusting for potential confounders, including age, mesh type, and other unbalanced baseline variables, our model indicated that hysterectomy was associated with a nearly tenfold higher risk of mesh exposure. This conclusion is similar to those of previous studies. In the CARE trial, which included 322 women undergoing SC with complete 2-year follow-up, concurrent hysterectomy was associated with a fivefold increase in the risk of mesh/suture erosion compared with those without hysterectomy [8]. In this study, SC was performed via the open approach. In another single-center prospective study, Illiano et al. compared the outcomes in women who underwent LSC with or without hysterectomy with a median follow-up of 65.3 months, and they found that the anterior and posterior success rates of the hysterectomy group were higher than those of the uterine preservation group and that the mesh exposure rate was higher in the hysterectomy group (6 out of 82, 7.3% vs 2 out of 54, 3.7%) [19]. However, there are also some studies that reached different conclusions than ours. Pan et al. retrospectively analyzed clinical data of 34 patients undergoing LSC and laparoscopic total hysterectomy and 65 patients undergoing laparoscopic sacrohysteropexy. They found no mesh exposure cases in the two groups after a mean follow-up duration of 33 months, and the subjective satisfaction rate was higher in the hysterectomy group [20]. In their study, Pan et al. employed a method of placing the mesh without totally opening the posterior peritoneum and used a Y-shaped mesh instead of two-piece meshes, which may reduce the mesh exposure. Gagyor et al. compared the postoperative outcomes of women with concurrent total hysterectomy/supracervical hysterectomy and those with uterine preservation, and no significant differences in clinical and patient-reported outcomes were observed except for a lower anterior compartment failure in the uterine-sparing group [21]. Interestingly, they reported a higher postoperative mesh complication rate in the uterine-sparing group. which they attributed to superior mesh placement in patients undergoing concomitant hysterectomy. This may be related to the relatively greater technical difficulty of inserting the mesh in the uterine-sparing group than in the hysterectomy group. In our study, the rate of mesh exposure was higher in the hysterectomy group. We believe that this is related to the vaginal bacteria contamination introduced by the opening of the vaginal cuff [7].

Another point to note is that the mesh exposure rate in our study was higher than in previous studies [10, 22, 23]. Several factors may explain the increased mesh exposure risk. Primarily, the transvaginal placement of mesh in the hysterectomy group may have contributed to the risk of mesh exposure. Similar to our study, Tan-Kim et al. reported a high mesh exposure rate of 23% in a cohort involving vaginal attachment of the mesh [24]. In addition, the mesh weight has been identified as a crucial parameter. Results from a retrospective cohort study showed less mesh exposure when using the ultra-lightweight mesh [25]. However, in our cohort, more than two thirds of women used Gynemesh, a type of mesh weighing 42 g/m^2^. Furthermore, patients with mesh exposure were more likely to seek medical help owing to uncomfortable symptoms, potentially increasing the detection rate.

A recent meta-analysis, which included 9 observational studies, compared the efficacy outcomes of total or subtotal hysterectomy with those of uterine preservation during LSC, and reported that LSC with hysterectomy was associated with higher subjective and objective success rates [12]. However, in this study, we found no statistically significant difference in the success rates between the two groups. Although the postoperative POP-Q points Aa, C, Ap, and Bp showed statistical differences between the two groups, the anatomical success rates were high and no significant difference was observed.

Our study has several strengths. First, our data were sourced from a prospectively maintained database, enhancing the reliability of the recorded information. Beyond reporting incidence rates, we provided detailed clinical characterization of the mesh exposure events, including their location, size, management, and symptomatic presentation, which offers valuable insights for clinical management. Second, this study had a relatively long median follow-up duration, which is critical for capturing long-term complications. Finally, we used the validated questionnaires for patient-reported outcomes to ensure a standardized assessment of subjective symptoms.

Several limitations should also be acknowledged. First, the relatively small sample size resulted in a wide 95% CI for the OR, and future studies with a larger sample size are needed for more definitive estimates. Second, as a retrospective study, residual confounding may persist despite our statistical adjustments. It is necessary to further explore the issue through a randomized controlled trial, which has a higher level of evidence. Finally, the study was conducted in a single center. Although this promotes standardized surgical and follow-up protocols, it may limit the generalizability of the findings.

In conclusion, this study found that concomitant hysterectomy during LSC was associated with a significantly increased risk of mesh exposure. However, this risk needs to be balanced against the potential benefits of hysterectomy and the future surgical risks of uterine preservation. Therefore, we suggest that the decision should be individualized through careful risk–benefit assessment and thorough shared decision-making with patients.

## Supplementary Information

Below is the link to the electronic supplementary material.

Fig. 1 (PNG 36 KB)

(TIF 259 KB)

Fig. 2 (PNG 62 KB)

(TIF 469 KB)

## Declarations

The study was approved by the Ethics Committee of Peking Union Medical College Hospital (No. K5936)
